# Stereochemical and Structural Characterization of Methionine Oxidation in the IgG1 Fc Region by Integrated NMR and LC-MS Analysis

**DOI:** 10.1021/acs.analchem.5c06092

**Published:** 2026-02-11

**Authors:** Maho Yagi-Utsumi, Saeko Yanaka, Noritaka Hashii, Kohei Tomita, Takashi Misawa, Yosuke Demizu, Akiko Ishii-Watabe, Koichi Kato

**Affiliations:** † Exploratory Research Center on Life and Living Systems (ExCELLS), 597025 National Institutes of Natural Sciences, Okazaki, Aichi 444-8787, Japan; ‡ Core for Spin Life Sciences, Okazaki Collaborative Platform, National Institutes of Natural Sciences, Okazaki, Aichi 444-8787, Japan; § Institute for Molecular Science, National Institutes of Natural Sciences, Okazaki, Aichi 444-8787, Japan; ∥ Faculty and Graduate School of Pharmaceutical Sciences, 692588 Nagoya City University, Nagoya, Aichi 467-8603, Japan; ⊥ Materials and Structures Laboratory, Institute of Integrated Research, Institute of Science Tokyo, Yokohama, Kanagawa 226-8503, Japan; # National Institute of Health Sciences, Kawasaki, Kanagawa 210-0821, Japan

## Abstract

Monoclonal antibodies are widely used biotherapeutics, whose efficacy and pharmacokinetics critically depend on their structural integrity. Among chemical degradation pathways, methionine oxidation is a particularly important post-translational modification that compromises antibody stability, Fc receptor binding, and thereby FcRn-mediated recycling and FcγR-mediated effector functions. However, the structural consequences of oxidation remain poorly understood, largely due to the subtle and localized nature of the modification. Here, we present an integrated analytical framework combining methyl-based NMR spectroscopy, selective enzymatic reduction, and peptide mapping to resolve methionine oxidation in the Fc region of human IgG1 antibodies at residue- and stereochemical-level resolution. By selectively labeling methionine methyl groups, we monitored oxidation-induced spectral changes in conserved Fc residues Met252 and Met428. Site-directed mutagenesis revealed a mutual influence between these residues, consistent with their spatial proximity at the C_H_2–C_H_3 domain interface. Stereospecific reduction with methionine sulfoxide reductase A enabled the assignment of R- and S-isomers, while peptide mapping by liquid chromatography–mass spectrometry corroborated the NMR findings. This combined approach demonstrated that Met252, which is solvent-exposed, is more susceptible to oxidation than buried Met428 and that both residues display stereochemical heterogeneity that modulates local structure. By bridging chemical modifications and higher-order structural perturbations, this integrated framework provides mechanistic insights into how methionine oxidation impairs antibody function. More broadly, it establishes a basis for quality assurance and rational design of therapeutic antibodies with improved stability.

## Graphical abstract

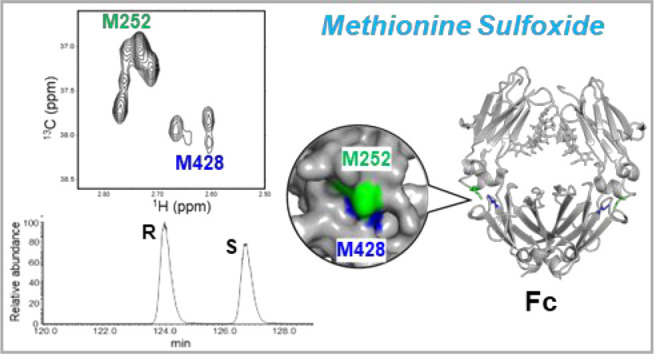

## Introduction

Monoclonal antibodies are among the most widely used biotherapeutics, with applications spanning oncology, immunology, and infectious diseases.^1,2^ Their clinical efficacy and pharmacokinetic behavior depend not only on antigen specificity but also on the structural integrity of the antibody molecule.^3^ Among various chemical degradation pathways, methionine oxidation, particularly at conserved Fc domain residues Met252 and Met428 in human IgG1, is a critical post-translational modification that can compromise antibody stability, Fc receptor binding, and thereby FcRn-mediated recycling and FcγR-mediated effector functions such as antibody-dependent cellular cytotoxicity.^4^

The functional consequences of methionine oxidation have been well documented. Stracke et al. demonstrated that oxidation at Met252 disrupts interaction with the neonatal Fc receptor (FcRn), resulting in accelerated systemic clearance of IgG1.^5^ Glover et al. showed that metal-catalyzed oxidation, driven by trace amounts of Cu­(II) or Fe­(II), induces site-specific oxidative stress.^6^ This leads to aggregation, fragmentation, and loss of biological activity, even when antigen binding remains unaffected.

Despite its relevance, the structural consequences of methionine oxidation remain difficult to assess due to the subtle and localized nature of the modification.^5^ Conventional analytical techniques such as mass spectrometry and peptide mapping provide residue-level chemical information but offer limited insight into higher-order conformational changes or stereochemical diversity.

Nuclear magnetic resonance (NMR) spectroscopy offers a unique advantage in this context. In particular, methyl-based NMR methods, such as ^1^H–^13^C correlation spectroscopy, allow sensitive monitoring of higher-order structural changes at the atomic level. Indeed, NMR has begun to be used for the detection of methionine oxidation in therapeutic antibodies,^7–11^ though signal assignment and stereochemical resolution remain challenging.

To address these limitations, we developed an integrated analytical approach combining methyl-based NMR spectroscopy, selective enzymatic reduction, and peptide mapping to investigate methionine oxidation in the Fc region of human IgG1 antibodies. Our aim was to interpret complex spectral changes associated with oxidation-induced local structural perturbations at the amino acid residue level. By focusing on the conserved residues Met252 and Met428, which are key sites implicated in FcRn binding and antibody stability, we sought to establish a generalizable framework for evaluating oxidation at atomic resolution and to provide insights relevant to the design and optimization of therapeutic antibodies. By elucidating the relationship between chemical modifications such as methionine oxidation and higher-order structural perturbations, this study also contributes to ensuring the structural integrity and quality of therapeutic antibodies.

## Experimental Section

### Expression and Purification of Wild-Type and Mutant Fc

The mammalian cell expression system was used to produce the Fc region of IgG1 (wild type, WT) and two Fc mutants (M252V and M428V). Plasmids encoding the Fc WT and mutants in the pcDNA3.4 vector were transfected into Expi293 cells using the Gibco Expi293 Expression System Kit (Thermo Fisher Scientific). Transfection and culture conditions were carried out according to the kit protocol.

For ^13^C-labeling of the methyl group of methionine side chains, cells were grown in medium containing l- [methyl-^13^C]­methionine. Around 1 week after transfection, when cell viability declined, the culture was centrifuged, and the supernatant was subjected to affinity purification using a protein G column (Cytiva, Tokyo, Japan) equilibrated with PBS (pH 7.0). Bound Fc glycoproteins were eluted with glycine buffer and immediately neutralized. Further purification was performed by size exclusion chromatography on a Superdex 200 HiLoad 16/600 column (Cytiva) equilibrated with PBS.

### Oxidation of Fc

Purified Fc samples were oxidized by incubation with either 5% *tert*-butyl hydroperoxide (tBHP) or 0.01–10% (v/v) hydrogen peroxide (H_2_O_2_) for 3 h at 37 °C. The oxidation reaction was quenched by buffer exchange into PBS containing methionine (final concentration: 15 mM) using an Amicon^Ⓡ^ Ultra centrifugal filter unit, repeating the exchange three times.

### Selective Reduction of Methionine Sulfoxide in Fc

Recombinant methionine sulfoxide reductase A (MsrA) from *S. cerevisiae*, an enzyme that specifically reduces the S-isomer of methionine sulfoxide, was prepared according to previous reports.^12^ MsrA was expressed with an N-terminal His_6_-tag using the pET28b vector in *E. coli* BL21­(DE3) and purified using a Ni^2+^-immobilized affinity column (cOmpleteTM His-Tag Purification Resin, Roche) from the soluble lysate. Then, MsrA was purified using a HiLoad 16/600 Superdex 200 pg column (Cytiva) equilibrated with 25 mM Tris-HCl, 100 mM NaCl, 10 mM imidazole, 1 mM EDTA, 1 mM dithiothreitol (DTT), and 1 mM phenylmethylsulfonyl fluoride (PMSF).

Oxidized WT and mutant Fc samples were reduced by incubating them with 1 μM MsrA in the presence of 10 mM DTT at 25 °C for 5 h, using 20 μM oxidized Fc as the substrate.

### NMR Measurements

The Fc samples were buffer-exchanged into 5 mM sodium phosphate buffer (pH 7.4) containing 50 mM NaCl in 99.9% D_2_O using an Amicon^Ⓡ^ Ultra centrifugal filter unit.

Two-dimensional ^1^H–^13^C XL-ALSOFAST–HMQC spectra^13^ were recorded at 42 °C on an AVANCE 800 spectrometer equipped with a cryogenic probe (Bruker BioSpin). The spectra were acquired at a ^1^H observation frequency of 800 MHz with a spectral width of 30 ppm in the ^13^C dimension (*F*_1_) and 12 ppm in the ^1^H dimension (*F*_2_). The size of the FID was set to 302 complex points in *F*_1_ and 1920 complex points in *F*_2_, with 80 scans per *t*_1_ increment. The relaxation delay (*d*_1_) was set to 1000 ms, and the INEPT delays (τ_1_ and τ_2_) were 2.7 and 2.2 ms, respectively. The ^13^C carrier frequency was centered at 20 ppm for the unoxidized methionine and 30 ppm for the methionine sulfoxide. All NMR data were processed using TopSpin (Bruker, Billerica, MA) and Sparky (Lee et al., 2015).

### LC-MS Analyses

The sample preparation procedure and liquid chromatography–mass spectrometry (LC-MS) analytical conditions for peptide mapping were in accordance with our previous report.^14^ The tryptic peptides containing Met428 sulfoxide R-form or S-form were identified by comparing with synthetic oxidized peptides. The oxidation levels and the ratio of R:S forms for Met428 sulfoxide were manually calculated using peak area obtained by full mass scan analysis. For an accurate calculation of the ratio of R:S forms, the two diastereomeric peptides were completely separated using a linear gradient from 5% B to 10% B for 140 min.

## Results and Discussion

### NMR Spectral Changes upon Methionine Oxidation

In this study, we prepared Fc fragments selectively labeled at methionine methyl groups with ^13^C via metabolic labeling and subjected them to XL-ALSOFAST-HMQC measurements. We previously completed comprehensive assignments of all methyl peaks in IgG1-Fc, including those from Met252 and Met428.^15,16^ In this study, we specifically focus on the two methionine methyl peaks shown in Figure 1A. Upon treatment with 5% tBHP at 37 °C for 3 h, these peaks diminished in intensity and gave rise to new signals with distinct chemical shifts within the typical methyl region of unoxidized methionine (^1^H: 1.9–2.2 ppm; ^13^C: 13.5–16.0 ppm) (Figure 1B). Concurrently, signals attributable to methionine sulfoxide methyl groups emerged, each presenting as multiple peaks (^1^H: 2.5–2.8 ppm; ^13^C: 36.5–38.5 ppm). Treatment with 10% H_2_O_2_ under the same conditions led to complete disappearance of the unoxidized methionine peaks, while the methionine sulfoxide signals still appeared as multiple peaks, albeit with altered intensity ratios (Figure 1C).

**Figure 1:**
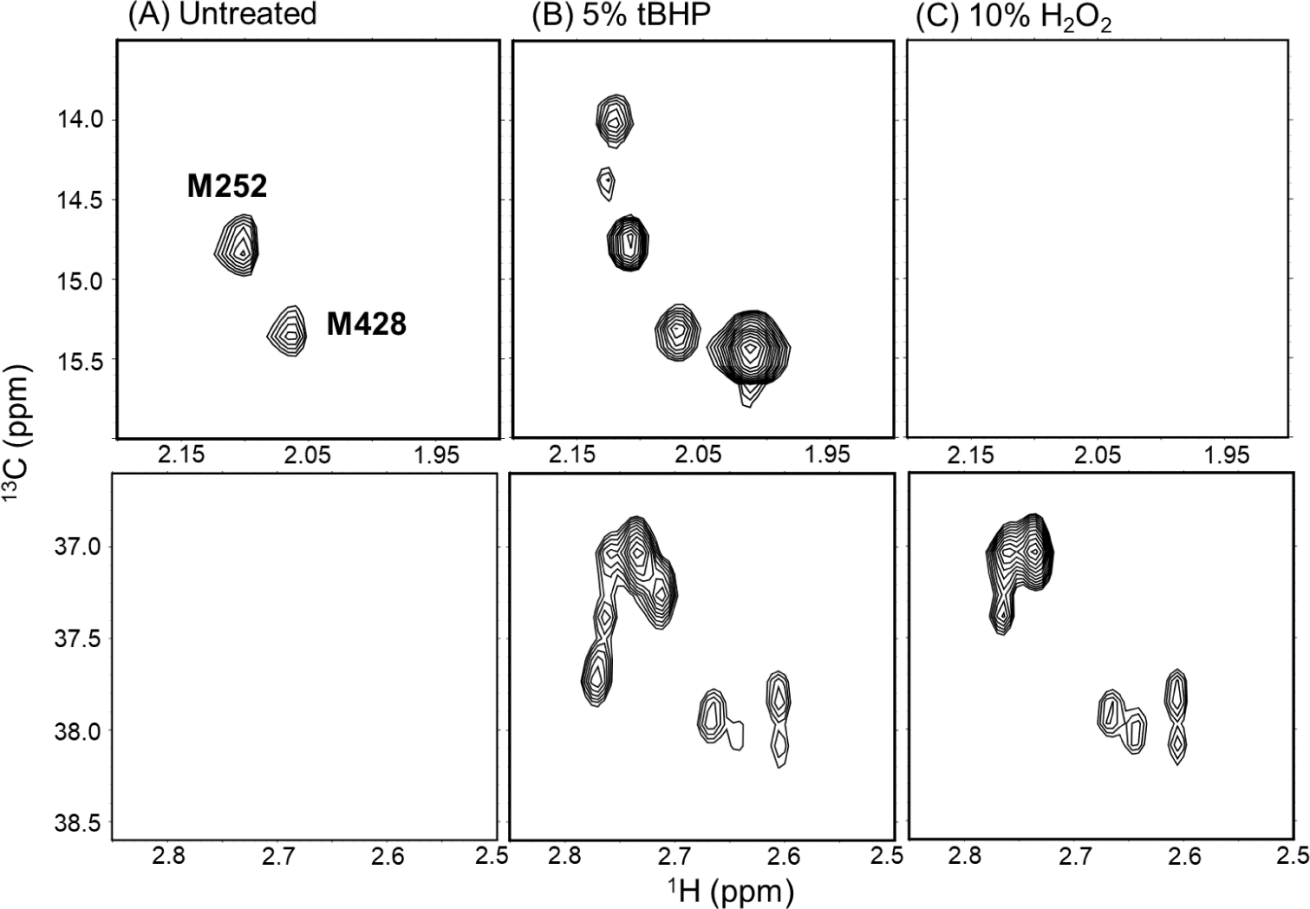
NMR spectral changes of methionine methyl groups in IgG1 Fc upon oxidation. ^1^H–^13^C XL-ALSOFAST-HMQC spectra of IgG1 Fc selectively labeled with ^13^C at methionine methyl groups: (A) untreated, (B) treated with 5% tBHP, and (C) treated with 10% H_2_O_2_. The original unoxidized methionine signals decreased upon tBHP treatment, accompanied by the appearance of new peaks attributed to unoxidized methionine residues experiencing altered local environments (B, upper), as well as peaks corresponding to methionine sulfoxide formation (B, lower). Treatment with 10% H_2_O_2_ led to complete loss of unoxidized signals, while sulfoxide peaks remained as multiple signals with altered intensity ratios (C, lower).

### Spectral Changes in Methionine-Substituted Mutants

To selectively observe signals from individual methionine residues, we generated Fc mutants in which one of the two methionine residues was substituted with valine. Spectral analysis revealed that substitution of one methionine affected the chemical shift of the remaining methionine peak, indicating mutual influence between Met252 and Met428 (Figure 2A). This shift perturbation is consistent with their spatial proximity at the C_H_2–C_H_3 domain interface of the Fc. Upon H_2_O_2_ treatment of each mutant, the unoxidized methionine peaks decreased in intensity without shifting, while the resulting methionine sulfoxide signals appeared as two distinct peaks in both mutants (Figure 2B). This indicates that the heterogeneity of the sulfoxide peaks observed in the wild-type spectrum arises partly from interactions between the two methionine (or sulfoxide) residues, and partly from the intrinsic ability of each oxidized methionine to adopt two distinct states.

**Figure 2:**
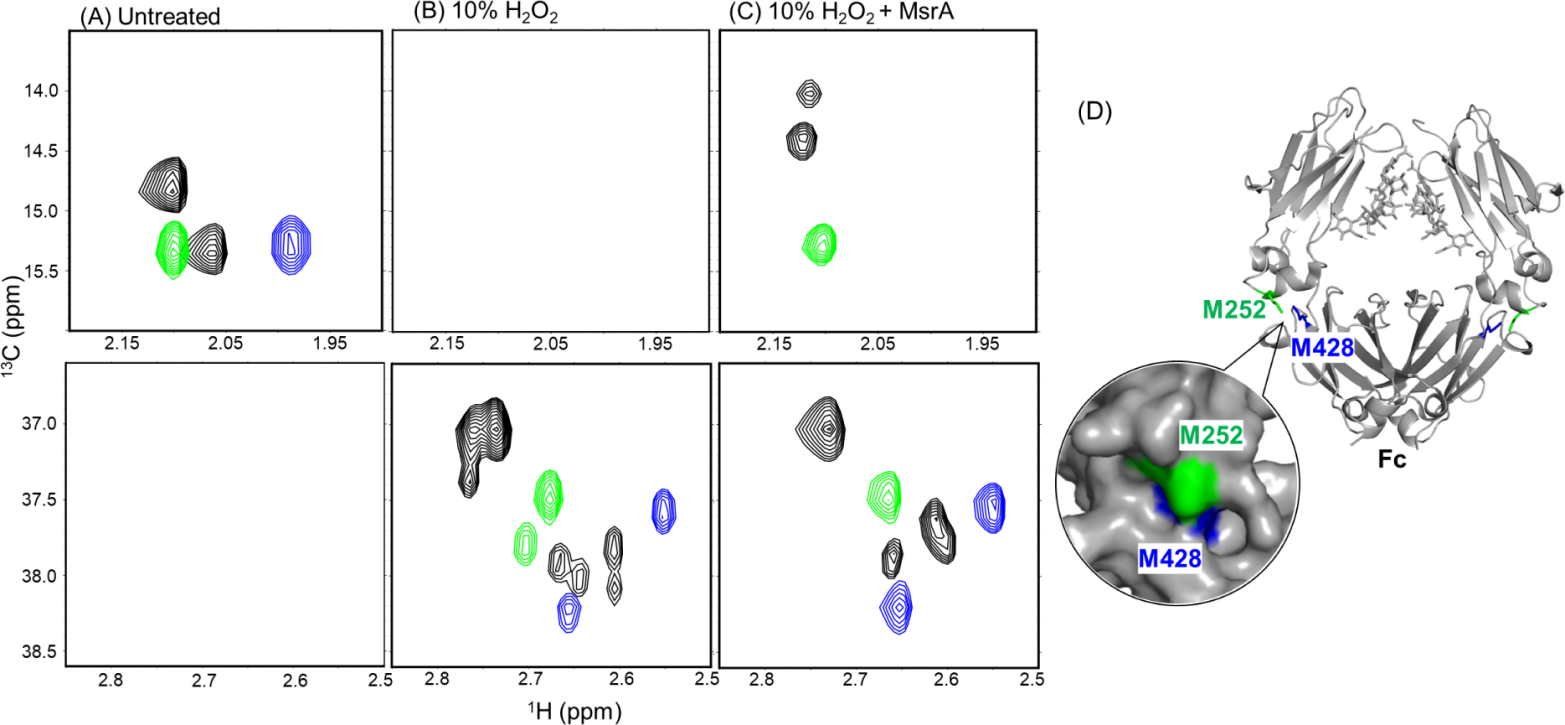
NMR spectral signatures of methionine-substituted IgG1 Fc mutants compared with WT Fc. ^1^H–^13^C XL-ALSOFAST-HMQC spectra of IgG1 Fc WT (black), M252V mutant (blue), and M428V mutant (green). (A) Untreated, (B) after incubation with 10% H_2_O_2_, and (C) after treatment with 10% H_2_O_2_ followed by MsrA. Spectra are shown for the unoxidized methionine region (upper) and methionine sulfoxide region (lower). In (B), upon H_2_O_2_ treatment, unoxidized signals decreased while sulfoxide peaks appeared. In (C), MsrA selectively reduced the S-form of methionine sulfoxide in WT and the M428V mutant (corresponding to Met252). (D) Structural model of the Fc region (PDB: 3AVE)^18^ highlighting Met252 (green) and Met428 (blue), indicating their spatial proximity. Met252 is solvent-exposed, whereas Met428 is buried within the C_H_2–C_H_3 domain interface.

### Spectroscopic Characterization of Reductase-Induced Changes

Methionine sulfoxide side chains exist as stereoisomers: R and S forms. Methionine sulfoxide reductases comprise two classes, MsrA and MsrB, which selectively reduce the S- and R-forms of methionine sulfoxide, respectively;^12,17^ here, MsrA was used to enable stereochemical discrimination in the NMR analysis. When MsrA was applied to wild-type and M428V mutant Fc samples oxidized with 10% H_2_O_2_, a subset of the sulfoxide peaks disappeared (Figure 2C). In contrast, no effect was observed in the M252V mutant, indicating that the spectral changes in the wild type were due to selective reduction of the Met252 sulfoxide S-form. This observation is consistent with the structural context: Met252 is solvent-exposed, whereas Met428 is buried, limiting its accessibility to the enzyme (Figure 2D). Consistent with this interpretation, the S-form of Met428 sulfoxide was not completely removed upon MsrA treatment, reflecting its limited accessibility to the enzyme.

Even after near-complete reduction of the Met252 sulfoxide S-form in the wild-type spectrum, the remaining sulfoxide peaks still exhibited spectral heterogeneity (Figure 2C). Interestingly, the restored unoxidized Met252 also gave rise to two resolved signals. These observations suggest that both Met252 and Met428 exist in three states—unoxidized, sulfoxide R-form, and sulfoxide S-form—and that the observed spectral heterogeneity arises from combinations of their own and their partner’s oxidation states. The mutual influence on chemical shifts is consistent with their spatial proximity at the C_H_2–C_H_3 domain interface of the Fc.

### Spectral Assignments

To assign the observed peaks and to establish the assignments summarized in Figure 3, we performed quantitative correlation analysis integrating NMR data with peptide mapping by LC-MS. The peak assignment proceeded through the following stepwise workflow. First, the signals of unoxidized Met252 and Met428 were anchored based on the spectra of untreated Fc and Met→Val mutants. The stereochemical identities of Met252 sulfoxide (R and S forms) were then established using MsrA, which selectively reduces the S-form of methionine sulfoxide (Figure S1), confirming that Met428 sulfoxide is inaccessible to this enzyme. Next, partially oxidized samples generated under gradually increasing H_2_O_2_ concentrations (0.01–0.2%) were used to track the sequential appearance and splitting of peaks in both the unoxidized and sulfoxide regions, reflecting the intrinsic precedence of Met252 oxidation over Met428. Finally, quantitative LC-MS measurements of R/S ratios were used as a boundary condition to distinguish Met428-R from Met428-S by comparing LC-MS-derived ratios with the total NMR signal intensity assigned to each Met428 isomer across all Met252 oxidation subclasses. Alternative assignments were rejected because they failed to satisfy both data sets simultaneously.

**Figure 3:**
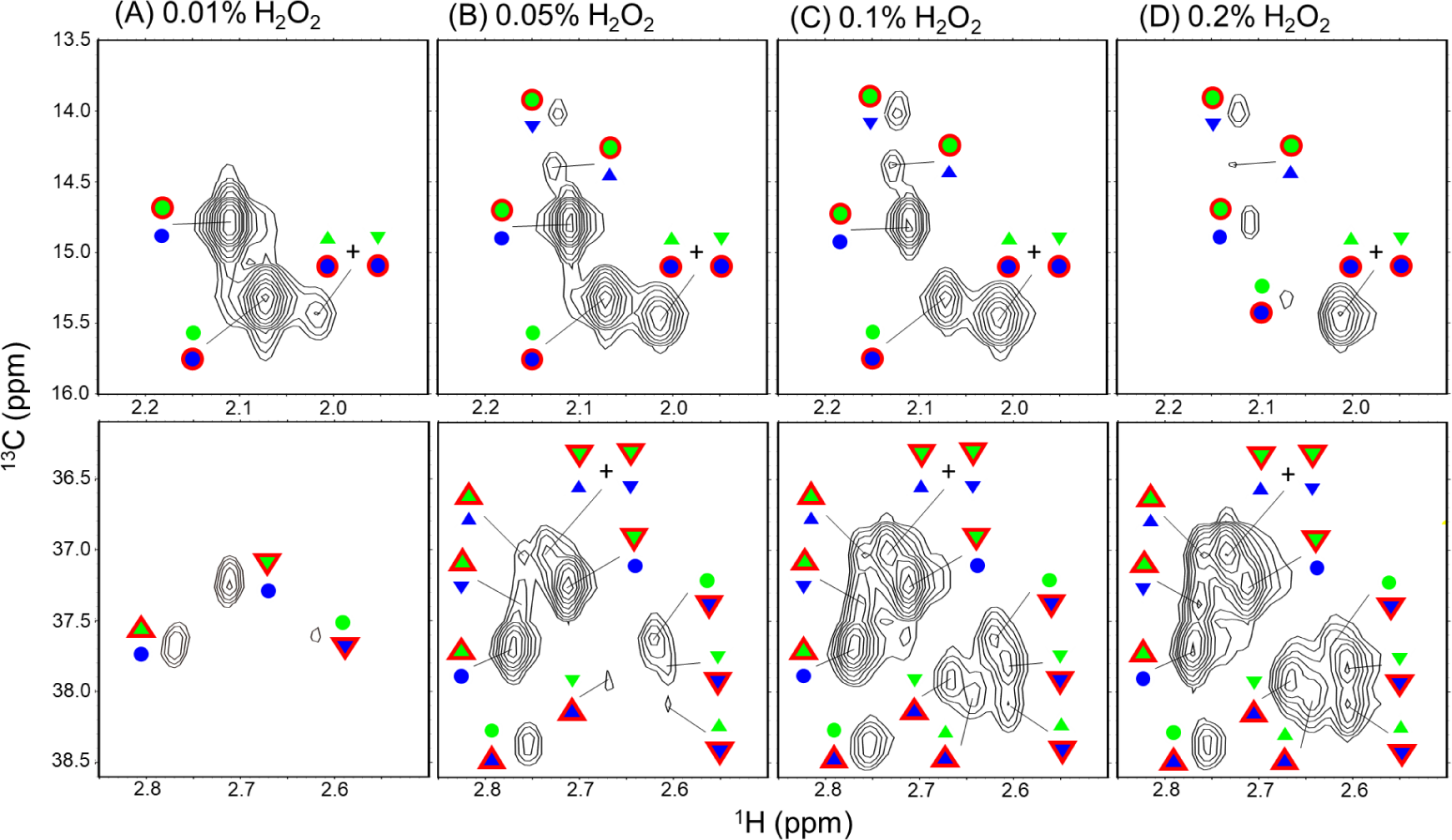
NMR spectral assignments of methionine and methionine sulfoxide signals in IgG1-Fc. ^1^H–^13^C XL-ALSOFAST-HMQC spectra of IgG1-Fc selectively labeled with ^13^C at methionine methyl groups after mild oxidation with H_2_O_2_: (A) 0.01%, (B) 0.05%, (C) 0.1%, and (D) 0.2%. Spectra are shown for the unoxidized methionine region (upper) and methionine sulfoxide region (lower). Observed NMR signals are annotated using a symbol-based assignment scheme. Assignments for Met252 are shown in green, and those for Met428 are shown in blue. Unoxidized methionine, methionine sulfoxide S-form, and methionine sulfoxide R-form are indicated by circles (○), upright triangles (△), and inverted triangles (▽), respectively. Symbols enclosed by a red outline denote experimentally observed peaks, whereas symbols without a red outline indicate the interaction partner whose oxidation or stereochemical state influences the observed peak position.

Based on this workflow, wild-type Fc was then treated with mild concentrations of H_2_O_2_ (0.01%, 0.05%, 0.1%, 0.2%) to generate partially oxidized samples, which were analyzed by both NMR and LC-MS.

Peptide mapping revealed that Met252 is more readily oxidized than Met428, consistent with previous reports and the differential solvent exposure of the two residues (Figure 4). Interestingly, a 23-residue peptide containing oxidized Met428 (WQQGNVFSCSVM^428^HEALHNHYTQK) yielded two distinct peaks, which are thought to reflect the stereochemical differences between the R- and S-forms of Met428 sulfoxide (Figure 5). This interpretation was supported by synthetic diastereomeric peptides: a mixture of Met428 sulfoxide R- and S-form peptides produced two corresponding peaks. Furthermore, the peptide synthesized with the S-form of Met428 sulfoxide gave a single peak at 14.1 min, allowing the 14.0 min peak to be assigned to the peptide containing the R-form. Stereochemical assignment of the methionine-sulfoxide-containing peptides was further validated by selective reduction with MsrA, which confirmed specific removal of the S-diastereomer (Figure S2). In contrast, peptides containing oxidized Met252 (DTLM^252^ISR) yielded a single LC-MS peak, even though NMR analysis indicated the presence of both R- and S-forms of Met252 sulfoxide. This suggests that LC-MS was unable to resolve the stereoisomers of Met252, likely due to minimal chromatographic differences between the two forms.

**Figure 4:**
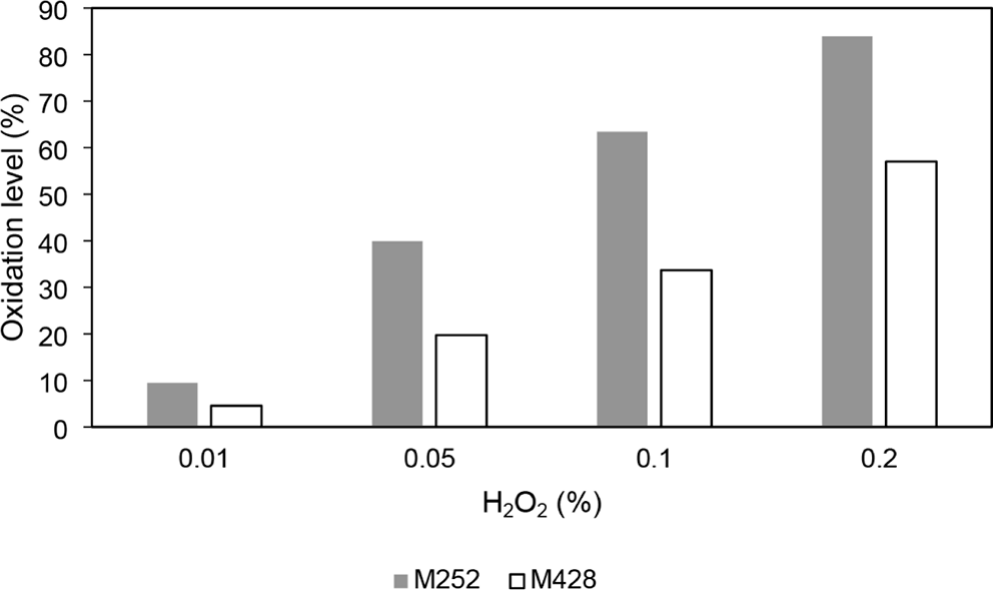
Comparative oxidation levels of M252 and M428 in the oxidized IgG1 Fc samples after treatment with H_2_O_2_ at concentrations of 0.01%, 0.05%, 0.1%, and 0.2%.

**Figure 5:**
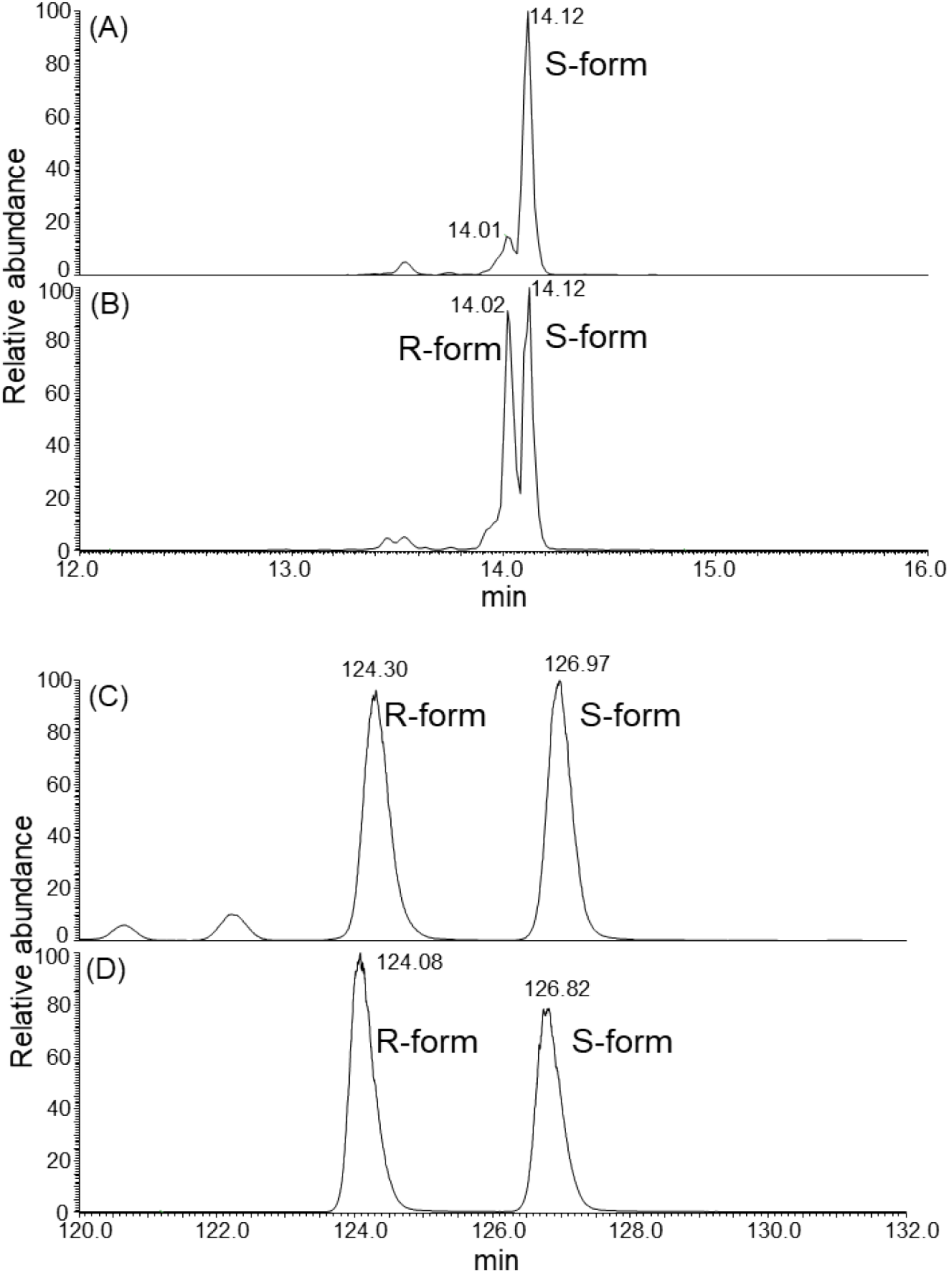
Extracted ion chromatograms (EICs) of [M+5H]^5+^ at *m*/*z* 564.35–566.16 corresponding to the peptide containing oxidized Met 428 (WQQGNVFSCSVM^428^HEALHNHYTQK) from IgG1 Fc. Panel A shows the synthetic peptide containing Met428 sulfoxide S-form. Panels B and C show the synthetic peptides containing Met428 sulfoxide R-form and S-form. Panel D shows tryptic digest of IgG1 Fc after treatment with 0.2% H_2_O_2_. Chromatographic conditions were: isocratic flow at 2% mobile phase B for 2 min and a linear gradient from 2% to 40% mobile phase B for 35 min (Panels A and B), and a linear gradient from 5% to 10% mobile phase B for 140 min (Panels C and D). LC-MS data were acquired using data-dependent scanning for Panels A and B, and single mass scanning for Panels C and D.

Across H_2_O_2_ concentrations, the ratio of R:S forms for Met428 sulfoxide remained consistent at approximately 55:45. Based on the relative ease of Met252 oxidation and the predominance of the R-form in Met428, we quantified NMR peak intensities and successfully assigned all methionine and methionine sulfoxide peaks in a manner consistent with LC-MS data (see Figure 3). While LC-MS analysis was unable to distinguish between the R- and S-forms of Met252 sulfoxide, NMR approach enabled estimation of an R:S ratio of 53:47 for Met252. This slight preference for the R-form, observed in both residues, likely reflects differences in their local microenvironments.

## Conclusion

In this study, we demonstrated that methyl-based NMR spectroscopy, integrated with selective enzymatic reduction and peptide mapping, enables residue-level and stereochemical resolution of methionine oxidation within the Fc region of IgG1 antibodies. By bridging the analytical gap left by conventional mass spectrometry and peptide mapping, our approach revealed how oxidation at Met252 and Met428 differentially affects local structure depending on stereochemistry and spatial context. These structural perturbations are directly linked to functional impairments, including reduced FcRn binding and altered pharmacokinetics, underscoring the need for oxidation analysis that accounts for both site specificity and stereochemical diversity. This integrated analytical framework thus provides a powerful basis for evaluating antibody integrity at atomic resolution and for guiding the rational design of more stable therapeutic proteins, while also establishing a foundation for quality assurance.

## Supplementary Material



## Notes

MsrA-treated wild-type Fc spectrum annotated for assignment verification (Figure S1); Validation of mass-chromatographic peak assignment for Met428 sulfoxide stereochemistry by MsrA treatment (Figure S2) (PDF)
