# Supplementary material for: Stereochemical and Structural Characterization of Methionine Oxidation in the IgG1 Fc Region by Integrated NMR and LC-MS Analysis
Source: Anal Chem. 2026 Feb 11;98(7):5316–22. doi: 10.1021/acs.analchem.5c06092 (PMC12937046; doi:10.1021/acs.analchem.5c06092)
Supplement: Supplementary file 1 [file ac5c06092_si_001.pdf]

Supporting Information

Stereochemical and Structural Characterization of Methionine Oxidation in the IgG1 Fc region by Integrated NMR and LC-MS Analysis

Maho Yagi-Utsumi[†,‡,§,||], Saeko Yanaka[†,‡,§,⊥], Noritaka Hashii[#], Kohei Tomita[||], Takashi Misawa[#], Yosuke Demizu[#], Akiko Ishii-Watabe[#], Koichi Kato[†,‡,§,||,*]

[†] Exploratory Research Center on Life and Living Systems (ExCELLS), National Institutes of Natural Sciences, Okazaki, Aichi 444-8787 Japan

[‡] Core for Spin Life Sciences, Okazaki Collaborative Platform, National Institutes of Natural Sciences, Okazaki, Aichi 444-8787 Japan

[§] Institute for Molecular Science, National Institutes of Natural Sciences, Okazaki, Aichi 444-8787 Japan

[||] Faculty and Graduate School of Pharmaceutical Sciences, Nagoya City University, Nagoya, Aichi 467-8603, Japan

[⊥] Materials and Structures Laboratory, Institute of Integrated Research, Institute of Science Tokyo, Yokohama, Kanagawa 226-8503, Japan

[#] National Institute of Health Sciences, Kawasaki, Kanagawa 210-0821, Japan

***Corresponding author:** Koichi Kato, ph.D.

kkatonmr@ims.ac.jp, 5-1 Higashiyama, Myodaiji, Okazaki 444-8787, Japan

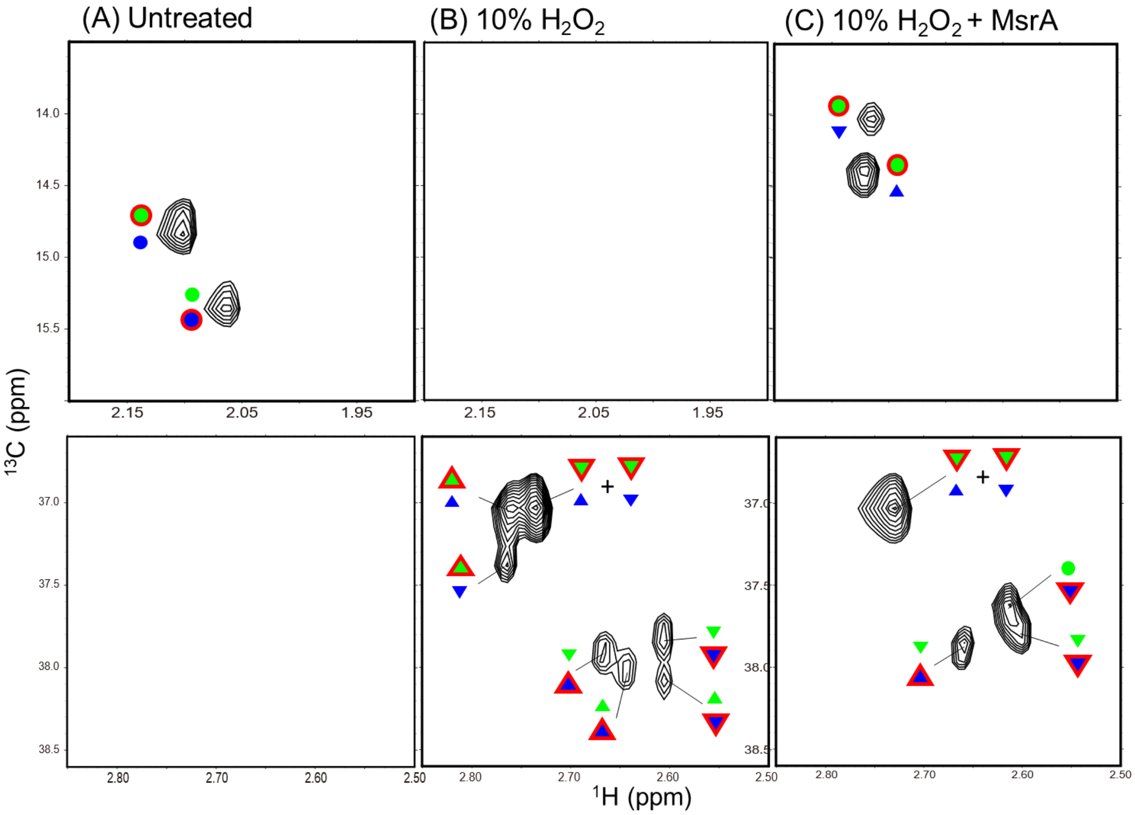

Figure S1. MsrA-treated wild-type Fc spectrum annotated for assignment verification.

^{1}H–^{13}C XL-ALSOFAST–HMQC spectra of IgG1 Fc WT. (A) Untreated, (B) after incubation with 10% H_2O_2, and (C) after treatment with 10% H_2O_2 followed by MsrA. Spectra are shown for the unoxidized methionine region (upper) and methionine sulfoxide region (lower). Signals corresponding to S-form Met252 sulfoxide and its neighboring perturbation disappear after MsrA treatment, while new unoxidized M252 peaks emerge (14.0 and 14.4 ppm). Partial persistence of the Met428 S-form is consistent with its buried location. This figure provides direct visual support for the assignment strategy used in Fig. 3.

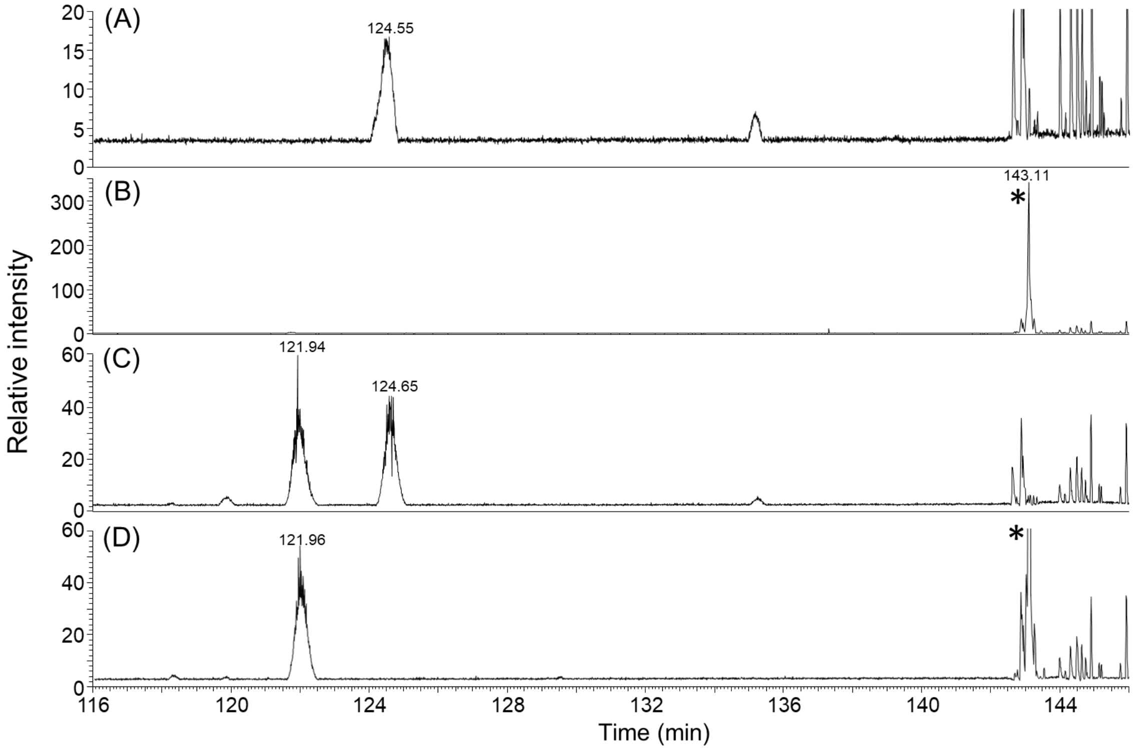

Figure S2. Validation of mass-chromatographic peak assignment for Met428 sulfoxide stereochemistry by MsrA treatment.

(A, B) Extracted ion chromatograms of the synthetic peptide (WQQGNVFSCSVM^{428}HEALHNHYTQK) containing the Met428 sulfoxide S-form before (A) and after (B) treatment with MsrA. (C, D) Extracted ion chromatograms of synthetic peptides containing a mixture of the Met428 R- and S-sulfoxide diastereomers before (C) and after (D) treatment with MsrA. Upon MsrA treatment, the signal corresponding to the S-form selectively disappeared, whereas the R-form remained unchanged. Peaks marked with an asterisk indicate species derived from the reduced (unoxidized) methionine form.
